# Detection and identification of enterohemorrhagic *Escherichia coli *O157:H7 and *Vibrio cholerae *O139 using oligonucleotide microarray

**DOI:** 10.1186/1750-9378-2-23

**Published:** 2007-12-23

**Authors:** Da-Zhi Jin, Xiao-Jing Xu, Su-Hong Chen, Si-Yuan Wen, Xue-En Ma, Zheng Zhang, Feng Lin, Sheng-Qi Wang

**Affiliations:** 1 Beijing Institute of Radiation Medicine, Beijing, 100850, China; 2 Wenzhou Medicine College affiliated Wenling First Hospital, Wenling, Zhejiang province, Wenling, 317500, China; 3 College of Animal Science and Medicine, Inner Mongolia Agricultural University, Hohhot, 010018, China; 4 Zhejiang Provincial Center for Disease Control and Prevention, Hangzhou, 310009, China

## Abstract

**Background:**

The rapid and accurate detection and identification of the new subtype of the pathogens is crucial for diagnosis, treatment and control of the contagious disease outbreak. Here, in this study, an approach to detect and identify *Escherichia coli *O157:H7 and *Vibrio cholerae *O139 was established using oligonucleotide microarray. We coupled multiplex PCR with oligonucleotide microarray to construct an assay suitable for simultaneous identification of two subtypes of the pathogens.

**Results:**

The *stx*1, *stx*2 gene and *uid*A gene having the specific mutant spot were chosen as the targets for *Escherichia coli *O157:H7, and meanwhile the *ctx*A, *tcp*A, and *LPSgt *gene for *Vibrio cholerae *O139. The oligonucleotide microarray was composed of eight probes including negative control and positive control from 16S rDNA gene. The six primers were designed to amplify target fragments in two triplex PCR, and then hybridized with oligonucleotide microarray. An internal control would be to run a PCR reaction in parallel. Multiplex PCR did not produce any non-specific amplicons when 149 related species or genera of standard bacteria were tested (100% specificity). In addition, *Escherichia coli *O157:H7 and *Escherichia coli *O157:non-H7, *Vibrio cholerae *O139 and *Vibrio cholerae *O1 had been discriminated respectively. Using recombinant plasmid and target pathogens, we were able to detect positive hybridization signals with 10^2 ^copies/μL and 10^3 ^cfu/mL per reaction.

**Conclusion:**

The DNA microarray assay reported here could detect and identify *Escherichia coli *O157:H7 and *Vibrio cholerae *O139, and furthermore the subtype was distinguished. This assay was a specific and sensitive tool for simultaneous detection and identification of the new subtype of two pathogens causing diarrhea in human.

## Background

*Escherichia coli *O157:H7 and *Vibrio cholerae *O139 are lethal pathogens capable of causing severe infectious diseases, characterized by acute outbreak, and large scale prevalence. As the statistics from Ministry of Health People Public of China shown [1], many incidents related to diarrhea due to two pathogens happened in recent year. The control and prevention means were extremely effective, since two kinds of new serotypes appeared. Meanwhile the government had taken many measures for studying detection and therapy assay. Therefore, we developed an assay for detecting the new serotypes of two pathogens. *E. coli *O157:H7 is capable of causing severe clinical symptoms such as enterohemorrhage and hemolytic uremic syndrome [2,3]. It can outbreak epidemically through the contamination of foods and water [4]. The pathogenicity of *E. coli *O157:H7 is related to various specific genes such as Shiga genes *stx1*, *stx2 *[5,6] and β-glucuronidase gene *uid*A (*gus*A) [7,8]. In some studies, *stx *genes were used to identify *E. coli *O157:H7 [9], but these genes existed in some O157: non-H7 serotypes, as well as some other types of pathogenic *E. coli *[10]. Therefore, mere detection of *stx *genes is not reliable for the purpose of identifying *E. coli *O157 serotype, and detection of specific genes to *E. coli *O157:H7 must be involved. The previous study also indicated that a strictly specific base mutation T93G occurs in the *uid*A gene of *E. coli *O157:H7 [6,11]. As a result, nucleic acid hybridization was used to detect this conserved mutation spot T93G of *uid*A gene in order to discriminate O157:H7 from other *stx*-producing intestinal bacteria.

*Vibrio cholerae *O139 is a strain of bacterium discovered in 1992 [12]. It can result in severe infectious diseases mainly accompanying with symptoms of diarrhea, characterized by acute outbreak, rapid spread, broad affected area, and large scale prevalence [13]. It was indicated that *Vibrio cholerae *O139 derived from *Vibrio cholerae *serotype O1 [14,15]. Both *Vibrio cholerae *O139 and *Vibrio cholerae *O1 have two principal virulence genes, *ctx*A and *tcp*A [16], yet they are different in the gene composition and sequence of *LPSgt *[17].

For a long time being, the traditional diagnosis of pathogenic *E. coli *O157:H7 and *Vibrio cholerae *O139 is mainly based on the conventional bacterial culture, which is a laborious and time-consuming procedure. It will take several days to obtain precise diagnostic results [18]. Therefore, it was very important to establish a rapid and specific method for detection and identification of pathogenic bacteria. Oligonucleotide microarray technology has been developed and widely used in the modern biology and life science research in recent years. It is featured by high throughput, low cost, and parallel detection of multiple genes simultaneously [9,19,20].

In the present study, a low-density oligonucleotide microarray was developed. Combined with multiplex PCR, virulence genes (*ctx*A, *tcp*A, stx1, stx2) and serotype specific genes (*LPSgt *for *Vibrio cholerae *O139 and *uid*A for *E. coli *O157:H7) are detected using the oligonucleotide microarray to detect and identify the pathogenic strains of *Vibrio cholerae *O139 and *E. coli *O157:H7 from clinical specimens simultaneously.

## Results

### Optimization of triplex PCR

**Figure 1:**
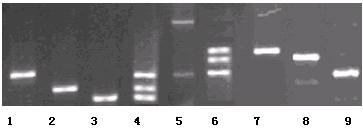


The triplex PCR was used to amplify the target gene segments in *E. coli *O157:H7 and *Vibrio cholerae *O139. In multiplex asymmetric PCR reaction, the variation of the ratio of forward primer to reverse primer and concentrations impacted the amplification efficiency obviously. Therefore, the signal intensities of hybridization were highly related to the ratio and amount of forward primer to reverse fluorescent primer for each gene. After the optimization, three PCR products in one tube had good specificity and average amplification efficiency. The concentration of primers was showed in Table 1, and then the result of agarose gel electrophoresis was shown in Figure 1.

**Table 1:** Triplex PCR primers for amplifying the target genes in *E. coli *O157:H7 and *Vibrio cholerae *O139

Target gene	Target organism	Sequence(5'-3')	PCR product length (bp)	Concentration in PCR mixture
*Stx*1	*E. coli *O157	F: GAA TTT ACC TTA GAC TTC TCG AC	250	0.15 μmol/L
		R: TCC TGT TAA CAA ATC CTG TCA C		0.75 μmol/L
*Stx*2	*E. coli *O157	F: TAC GAT AGA CTT TTC GAC TCA AC	207	0.1 μmol/L
		R: TCA ATA ATC AGA CGA AGA TGG TC		0.5 μmol/L
*Uid*A	*E. coli *O157:H7	F: TAA TGA GGA GTC CCT TAT GTT AC	179	0.1 μmol/L
		R: ACT GAT CGT TAA AAC TGC CTG G		0.5 μmol/L
*Ctx*A	*Vibrio cholerae*	F: ACT CAG ACG GGA TTT GTT AGG C	304	0.15 μmol/L
		R: ATC TAT CTC TGT AGC CCC TAT TAC		0.75 μmol/L
*Tcp*A	*Vibrio cholerae*	F: TTG ACC CAA GCA CAA TGT AAG AC	241	0.15 μmol/L
		R: CTA CTG TGA ATG GAG CAG TTC C		0.75 μmol/L
*LPS*gt	*Vibrio cholerae*	F: ACA TCT GTA GGG ATT GTA TTG AC	340	0.2 μmol/L
	O139	R: ATA ACA ACT GAG ATA TCA AGC GTC		1.0 μmol/L

### Screening of probes

**Figure 2:**
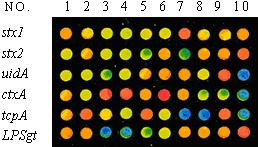


**Figure 3:**
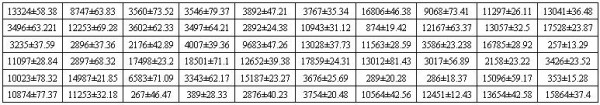


The 60 candidate probes were spotted in an array format of 6 rows × 10 columns. The PCR products labeled by fluorescence were mixed with hybridization solution and then hybridized with the probe array. The suitable probe for each gene was selected according to the specificity and hybridization signal intensity, which was analyzed by using GenePix 4.0 software program. The screening result of candidate probes was shown in Figure 2, and then the hybridization signal intensity corresponding to each candidate probe was shown in Figure 3. The statistical analysis of the quantified signal intensities indicated: *stx*1 No.7 probe, *stx*2 No.10 probe, *uid*A No.9 probe, and *LPSgt *No. 10 probe, had stronger signal intensity than that of other probes, at any same level of the PCR template amount, and in the meantime there are no non-specific signals. No.9 probe of *uid*A was a shorter probe (19 bp) containing +93 mutant spot in the middle of the sequence. With comparison to the No. 10 probe (without mutant point), this probe was strictly specific to the single base mutant *uidA *gene of *E. coli *O157:H7, indicating its applicability in detection of the *uid*A gene mutation spot. No.3, No.4, and No.6 probes for *ctx*A had statistically proximal signal intensities. Nevertheless, No.4 probe was more specific than the others. No.2, No.5, and No.9 probes for *tcp*A also had statistically proximal signal intensities at higher level of DNA template amount; whereas, No.9 probe had apparently higher signal intensities than No.2, No.5 probes at lower level of DNA template amount.

As a result, the optimal detecting probes for the six target genes of two pathogens were selected as follows: *stx*1 No.7 probe, *stx*2 No.10 probe, *uid*A No.9 probe, *ctx*A No.4 probe, *tcp*A No.9 probe, *LPSgt *No.10 probe.

**Figure 4:**
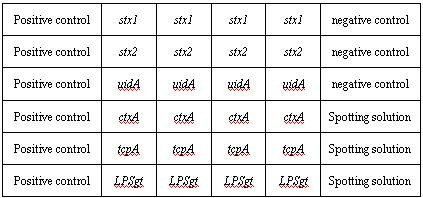


In addition, the bacterial universal probe was used as a positive control probes for verifying the PCR reaction was not inhibited by extraction impurities. And a plant gene segment with little sequence homology to the intestinal bacteria was chosen as a negative control probe. The final array format was shown as Figure 4. And the sequences of the optimal probe were listed in Table 2.

**Table 2:** Oligonucleotide probe sequences for identification of *E. coli *O157:H7 and *Vibrio cholerae *O139

Target gene	Sequence(5'-3')	Length (bp)	Tm(°C)^a^
Stx1	GTA CGT CTT TAC TGA TGA TTG ATA GTG GCA CAG GG	35	73.5
Stx2	AGT TAT TTT GCT GTG GAT ATA CGA GGG CTT GAT GT	35	69.6
uidA	TGG AAT TGA GCA GCG TTG G	19	70.1
ctxA	CAT ACA GTC CTC ATC CAG ATG AAC AAG AAG TTT CTG CTT TAG GTG	45	75.0
tcpA	CAG GAA GTG CCA ACT TAA ACC TAA CTA ATA TCA CGC ATG TTG AGA	45	76.8
LPSgt	TCG ATA AGA AGA GAT AAA GAT CTG AGT TAT CTA AAG ATA TTT G	43	71.2
Negative control	TCT TCG CCA GAG GCC TGC TAG CCT GGT TCA AGA TAC TAC C	40	68.5
Labeling control	Complimentary sequence of the reverse primer	20	70.5

^a^Tm(°C) was calculated using primer premier 5.0

### Specificity

The 149 strains of intestinal bacteria including various serotypes (parts as shown in Table 3) were detected under the optimal condition of triplex PCR and hybridization for testing the specificity of this oligonucleotide microarray. Two sets of triplex PCRs were performed to amplify bacterial DNA templates. Cy3 labeled PCR products were mixed and hybridized with the probe array fixed on the glass slides.

**Table 3:** part of the standard strains

Species	ATCC accession no^a^
*Escherichia coli *O157:H7	44752, 43889, 43859, W933, 882364, EDL 933
*Escherichia coli *O157:non-H7	S14–91, CB569, 5412, 493/89
*Escherichia coli *O55:H7	5905, 5A, 5B
*Escherichia coli *O26:H11	13C07, 13C08
*Escherichia coli *O111:NM	403, 405, TW00186
*Vibrio cholerae*	16025, 16026, 16028,
*Vibrio cholerae *O139	M045, 1837
*Vibrio cholerae *O1	569B, Ogawa
*Vibrio parahaemolyticus*	20502, 20506, 20507, 20511
*Enterobacter aerogenes*	CMCC (B) 45103,
*Proteus *spp	49027, 49101, 49102, 49103
*Yersinia enterocolitica*	52207, 52211, 52215, 52217, 52302
*Salmonella *spp	50001, 50004, 50013
*Clostridium botulinum*	64201, 64203
*Brucella *spp	23456, 11778
*Campylobacter jejuni*	33560, 7709, 29428, 43429
*Staphylococcus aureus*	26001, 26111, 26113,13565,27661
*Aeromonas hydrophila*	10501, 35654, 23211, 23213
*Citrobacter freundii*	CMCC (B) 48016
*Clostridium perfringens*	64711,13048
*Listeria monocytogenes*	54003, 54005, 54006, 54007
*Shigella *spp	51081, 3596, 451424
*Bacillus cereus*	63301,6051,63509
*Enterococcus faecalis*	32219, 32220, 32221, 32223
Species	ATCC accession no^a^
*Escherichia coli *O157:H7	44752, 43889, 43859, W933, 882364, EDL 933
*Escherichia coli *O157:non-H7	S14–91, CB569, 5412, 493/89
*Escherichia coli *O55:H7	5905, 5A, 5B
*Escherichia coli *O26:H11	13C07, 13C08
*Escherichia coli *O111:NM	403, 405, TW00186
*Vibrio cholerae*	16025, 16026, 16028,
*Vibrio cholerae *O139	M045, 1837
*Vibrio cholerae *O1	569B, Ogawa
*Vibrio parahaemolyticus*	20502, 20506, 20507, 20511
*Enterobacter aerogenes*	CMCC (B) 45103,
*Proteus *spp	49027, 49101, 49102, 49103
*Yersinia enterocolitica*	52207, 52211, 52215, 52217, 52302
*Salmonella *spp	50001, 50004, 50013
*Clostridium botulinum*	64201, 64203
*Brucella *spp	23456, 11778
*Campylobacter jejuni*	33560, 7709, 29428, 43429
*Staphylococcus aureus*	26001, 26111, 26113,13565,27661
*Aeromonas hydrophila*	10501, 35654, 23211, 23213
*Citrobacter freundii*	CMCC (B) 48016
*Clostridium perfringens*	64711,13048
*Listeria monocytogenes*	54003, 54005, 54006, 54007
*Shigella *spp	51081, 3596, 451424
*Bacillus cereus*	63301,6051,63509
*Enterococcus faecalis*	32219, 32220, 32221, 32223

It was shown that, 8 strains of *E. coli *O157:H7 had positive signal on *stx*1, *stx*2, and *uid*A probes. Two strains of *E. coli *O157: non-H7 had positive signals on *stx*1 and *stx*2 probes. Additionally, 3 strains of *E. coli *O157: non H7 and another serotype of *Escherichia coli *had signal on stx1 probe; 2 strains of *E. coli *O157: non H7 had signal on stx1 and stx2 probe, and furthermore *E. coli *O157: non-H7 had no signal on *uid*A probe. The results indicated that all the detected *E. coli *O157:H7 had both *stx*1 and *stx*2 toxin genes, whereas *E. coli *O157: non-H7 and other intestinal bacteria had one or both of the *stx*1 and *stx*2 toxin genes. However, the *uid*A probe can be used for identification of the O157:H7 from other intestinal bacteria accurately.

Seventy four strains of *Vibrio cholerae *O139 had positive signals on *ctx*A, *tcp*A, *LPSgt *probes; *Vibrio cholerae *(Ogawa and 569B) had positive signals on *ctx*A, *tcp*A probes. The results showed that *ctx*A, *tcp*A toxin genes may be detected in *Vibrio cholerae *O1 or O139 subtype. However, the probe specific to the unique region of *LPSgt *gene of *Vibrio cholerae *O139 was a strictly discriminating probe for *Vibrio cholerae *O139.

**Figure 5:**
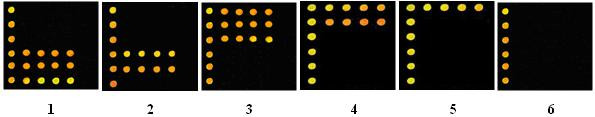


The fifty five strains of other intestinal bacteria and three non O1 non O139 *Vibrio cholerae *produces no detectable signal on the oligonucleotide microarray. The above results were shown in Table 4. Meanwhile, the hybridization images were shown in Figure 5, from which we could observe that the positive signals emerged orderly at the position corresponding to two pathogens from pure bacterial cultures; at the same time the signals of high intensity appeared at the position of positive control probes, and furthermore, no signals emerged at the position of negative control probes and blank probes.

**Table 4:** Test results of the 149 strains of intestinal bacteria using oligonucleotide microarray

Strains	Number	*stx*1	*stx*2	*uid*A	*ctx*A	*tcp*A	*LPS*gt
*E. coli *O157:H7	8(5.3%)	+	+	+	-	-	-
*E. coli *O157: non H7	2(1.3%)	+	+	-	-	-	-
*E. coli *O157: nonH7	3(2.0%)	+	-	-	-	-	-
EIEC	2(2.0%)	+	-	-	-	-	-
*Vibrio cholerae *O139	74(49.7%)	-	-	-	+	+	+
*Vibrio cholerae *O1	2(1.3%)	-	-	-	+	+	-
*Vibrio cholerae *Non O1 non O139	3(2.0%)	-	-	-	-	-	-
Other pathogens	55(36.9%)	-	-	-	-	-	-

^a ^American Type Culture Collection (ATCC) accession number

### Sensitivity

We chose 149 standard strains of intestinal bacteria including various serotypes for determining the cutoff value of each probe. A large number of results from hybridization with positive strains, negative strains and blank control indicated that the signal intensity of negative strains and blank control was greatly lower than that of positive strains. Thereby, cutoff value of the probe was the average signal intensity from negative bacteria and blank control plus 2SD. Based on the cutoff value corresponding to each probe (Table 5), the sensitivity of this DNA microarray assay was evaluated.

**Figure 6:**
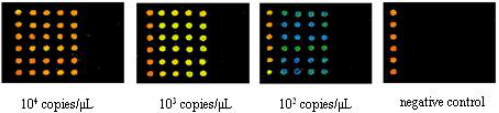


**Figure 7:**
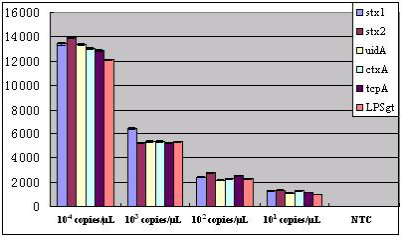


**Figure 8:**
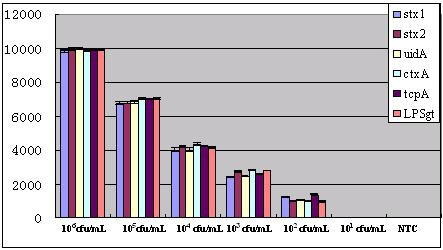


The recombinant plasmids were quantified and prepared in 10-fold serial dilutions, which were used as the PCR templates to test the sensitivity of the oligonucleotide microarray. In the triplex PCR mixture, when the template plasmids were 10^3^copies/μL, the PCR products can be detected both by using agarose gel electrophoresis and from the positive signal confirmed according to the cutoff value when hybridized with the detecting probe array; when the template plasmids were 10^2^copies/μL, the PCR products, which can be detected only by hybridizing with the detecting probe array (the ratio of signal to noise was greater than 4), produce no visible bands on the same agarose gel electrophoresis. Furthermore, when the template plasmids were 10 copies/μL, PCR products can not be detected either by using agarose gel electrophoresis or by hybridization with the detecting probe array. The above results and the value of signal intensity corresponding to serial dilution were shown in Figure 6 and in Figure 7 respectively. Furthermore, the test limit of real time PCR was 10^2 ^copies/μL(data not shown). Similarly when serial dilution (10-fold) of two targets from 10^6^~10^1^cfu/mL were evaluated, the positive signals were observed with a detection limit of 10^3^cfu/mL. The result of statistical analysis was shown in Figure 8. Meanwhile, the test limit of real time PCR and conventional microbiological methods was 10^3^cfu/mL and 10^2^cfu/mL respectively (data not shown).

**Table 5:** Cutoff value of the probe

Target gene	Species	Average of negative bacteria and blank control signal intensity	SD	Cutoff value^a^
ctxA	*Vibrio cholerae *O139	1783.12	64.27	1847.39
TcpA		1739.338	68.253	1807.591
LPSgt		1637.29	57.175	1694.465
stx1	*Escherichia coli*	1691.284	71.22	1762.504
Stx2	*O157:H7*	1790.385	78.473	1868.858
UidA		1802.1746	69.181	1871.3556
16S rDNA	internal control	1598.5629	24.75	1623.3129

^a ^cutoff value = average of negative bacteria and blank control signal intensity + 2 SD

### Detection of clinical specimens and double-blind mock samples

The clinical specimens were anus swabs collected from 60 patients in an epidemic *Vibrio cholerae *in *Zhejiang *Province, China. A total of 342 clinical specimens were detected by conventional microbiological methods, oligonucleotide microarray and fluorescent real-time PCR. The detection results were shown in Table 6.

**Table 6:** *Vibrio cholera *O139 testing result of the clinical specimens

Method	Target	Positive	Rate (%)
Conventional microbiological method	*Vibrio cholera *O139	89	26.0*
Oligonucleotide microarray	ctxA, tcpA, LPSgt	137	40.0
Fluorescent real time PCR	LPSgt	142	41.5

*: *p *< 0.05

It was shown that 89, 137, and 142 specimens were identified as *Vibrio cholerae *O139 with the methods of conventional culture, oligonucleotide microarray and fluorescent real-time PCR respectively. These results clearly indicated that the accuracy of oligonucleotide microarray was suitable for detection and identification of clinical specimen. Furthermore, through statistical analysis the *p *values (*p *< 0.05) were calculated in order to show that differences in the oligonucleotide microarray assay and conventional microbiological method were indeed significant in Table 6. The reason was that there were the target inactive organisms in the collected specimens in order that *Vibrio cholerae *O139 was detected positively using conventional culture.

Because most of clinical specimens collected were infected, we could not guarantee the validity of detecting *E. coli *O157:H7 in samples. Then, the 10 double-blind mock samples were prepared. From the results (Table 7) we observed that 8 of 10 mock samples were identified *E. coli *O157:H7 positive, and furthermore the above results were the same to double-blind results.

**Table 7:** *E. coli *O157:H7 testing result of the 10 double-blind mock samples

Sample No.	Oligonucleotide microarray test	Double-blind result
1	Negative	Negative
2	*E. coli *O157:H7	*E. coli *O157:H7
3	*E. coli *O157:H7	*E. coli *O157:H7
4	*E. coli *O157:H7	*E. coli *O157:H7
5	*E. coli *O157:H7	*E. coli *O157:H7
6	Negative	Negative
7	*E. coli *O157:H7	*E. coli *O157:H7
8	*E. coli *O157:H7	*E. coli *O157:H7
9	*E. coli *O157:H7	*E. coli *O157:H7
10	*E. coli *O157:H7	*E. coli *O157:H7

## Discussion

Oligonucleotide microarray, based on multiplex PCR reaction, could simultaneously detect multiple target genes of a pathogen, and therefore identify pathogens accurately. An oligonucleotide microarray containing six probes was established in the present study, capable of identifying Enterohemorrhagic *Escherichia coli *O157:H7 and *Vibrio cholerae *O139 at the same time. The reliability of oligonucleotide microarray to identify a specific serotype of bacterium from other types of bacteria with the highly homologous origin was closely depended on the specificity of probe to the "genetic marker" in the discriminating gene. In this study, *uid*A and *LPS*gt were selected as the discriminating genes, and the T+93G spot mutation in *uid*A gene specific to *E. coli *O157:H7 and the unique region in *LPS*gt gene specific to *Vibrio cholerae *O139 were selected as the genetic markers. After screening the candidate probes with bacteria with negative or positive presence of the target genes, 6 probes were selected with the best specificity and sensitivity.

It is crucial for a suitable detection assay to avoid false negative and false positive results efficiently. Then in this article, we chosen 16S rDNA gene as an internal control referred to previous study in order to confirm the genomic DNA was present and the process of PCR was not affected by the inhibitors and then a segment with little sequence homology to the intestinal bacteria was chosen as a negative control probe. When two duplex PCR reactions carried out, we ran a parallel reaction for amplifying a universal 500 bp fragment from 16S rDNA gene. The hybridization images showed that the signals of high intensity appeared at the position of positive control probes for every PCR and hybridization except negative control reaction and no any signals appeared at the position of negative control probes and blank probes. Accordingly, the results illustrated that the detection results obtained from this DNA microarray assay were reliable and accurate.

The value of cutoff was used to evaluating the result obtained from DNA microarray. In this paper, we had determined the cutoff value of each probe by trial and error. As the above results shown, based on the cutoff value corresponding to each probe, this oligonucleotide microarray could detect 10^2 ^copies of plasmid DNA templates in the triplex PCR reaction and 10^3 ^cfu/mL of target pathogens. For specimen with lower amount of bacteria than the detection limit, such as *E. coli *O157:H7 having minimal infective dose as low as 10 cells of bacteria, the step of bacterial cultivation and enrichment prior to PCR amplification was prerequisite for the purpose of avoiding the false-negative results. The detection accuracy of the oligonucleotide microarray established in this study was obviously higher than that of the conventional microbiological method and at a proximal level as that of the real-time PCR assay. However, compared with real time PCR, the oligonucleotide microarray can detect the major toxin genes that contribute to the severity of the infectious diseases as well as the unique gene regions specific to the *E. coli *O157:H7 *uid*A and *Vibrio cholera *O139 *LPS*gt. Meanwhile, the above results also showed that the sensitivity of this assay was lower than that of the conventional microbiological method. However the culture method cost more time and operation steps, and furthermore the sensitivity of DNA microarray, 10^3 ^cfu/mL, was sufficient to detect clinical specimen.

As we known, the oligonucleotide microarray was more sensitive than that of PCR assay. The main reason was that the result of PCR was identified using agarose gel electrophoresis, which limited the sensitivity of PCR. However, the results of detection of clinical specimens indicated that the sensitivity of oligonucleotide microarray was similar to that of real-time PCR. This result was reasonable because the principle of real time PCR contained PCR and hybridization with specific probe.

As it is very difficult to choose PCR fragments with different size for target pathogens and optimize and establish a stable and reproducible multiplex PCR and real-time PCR, the oligonucleotide microarray based on multiplex PCR could produce more diagnostic information than the real-time PCR. It can detect multiple pathogens and multiple target genes simultaneously in one assay.

In conclusion, a rapid and reliable DNA microarray assay was developed for detecting and identifying *E. coli *O157:H7 and *Vibrio cholera *O139 simultaneously. The specific probes fixed on the slide were chose from the *stx*1, *stx*2, and *uid*A genes for *E. coli *O157:H7 and the *ctx*A, *tcp*A, *LPSgt *genes for *Vibrio cholera *O139 respectively. Furthermore, this DNA microarray could distinguish from *E. coli *O157:H7 and *E. coli *O157: non-H7, *Vibrio cholera *O139 and *Vibrio cholera *non-O139. Then, the above results obviously indicated that the assay described here was applied to epidemiological study, clinical diagnosis, surveillance of food and public health, control of infectious diseases. Meanwhile, this study was a commence fabricating a DNA microarray for detecting and identifying many genera or species of pathogens, and we are adding more genera or species of bacteria including food borne pathogen and acute human pathogenic microorganisms to this detection system in order to increase the range, accuracy and discriminatory power of simultaneous detection.

## Methods

### Bacterial strains and genomic DNA isolation from the anus swabs

The standard strains of *E. coli *O157:H7 and *Vibrio cholerae *O139 were provided by the National Institute for the Control of Pharmaceutical and Biological Products of China. The local standard strains of *Vibrio cholerae *O1 (569B), *Vibrio cholerae *Ogawa, *Vibrio cholerae *O139 and *E. coli *O157:H7 and the standard strains of some common intestinal bacteria and the clinical specimens were provided by the Center for Disease Prevention and Control of *Zhejiang *province, China. Part of the standard strains was listed in Table 3. The pathogens that isolated from clinical specimens were identified by the conventional microbiological methods [21,22]. The double-blind 10 mock samples infected by *E. coli *O157:H7 were prepared. The genomic DNA from clinical samples was extracted by centrifugation as previously described [23]. Meanwhile DNA of the standard strains was extracted with UNIQ-10 column Genomic DNA Minipreps Kit (Sangon Co., LTD *Shanghai*, China), according to the manufacturer's instructions. Purified genomic DNAs were stored at -20°C in TE.

### Primers and probes

Based on references and published gene sequences in GenBank, *stx*1, *stx*2 and *uid*A genes were selected for *E. coli *O157:H7, *ctx*A, *tcp*A, *LPSgt *genes for *Vibrio cholerae *O139. Various sequences of each gene were collected and aligned by software Vector NTI Suite 9/AlignX. Primers and probes were designed using software Primer 5.0. The specificity of sequences was analyzed by BLAST GenBank. The 10 candidate probes were designed for each gene (totally 60 candidate probes), 19–60 mer in length. The Tm values of six primers and according to probes were approximately identical, and then the length of each PCR product was less than 500 bp. 16S rDNA gene was chosen as an internal control for verifying that genomic DNA templates of pathogens were present and the PCR reaction carried out successfully. Based on the previous study, a 500-bp fragment was obtained through PCR amplification using 16S-F1 (CGCTGGCGGCAGGCCTAACACATGC) and 16S-R1 (CGCGGCTGCTGGCACGGAGTTAGCC), and then the bacterial universal probe (ACTGAGACACGGTCCAGACTCCTACGGGAGGCAGCAGTAGGGAATATTG) was chosen as a marker for checking validity of internal control [24]. The oligonucleotide probes were modified with amino residue at 3' end. The reverse primer was labeled with fluorescent dye Cy3 at 5' end. The sequences of primers and probes were shown in Table 1 and Table 2 respectively.

### PCR amplification

A volume of 20 μL conventional PCR mixture contains: 0.1 μmol/L the forward primer, 1.0 μmol/L the reverse primer, 100 μmol/L dNTPs, 50–100 ng DNA template, 1× PCR buffer and 1.5 U *Taq *polymerase (Promega). PCR products were amplified in a thermal cycler (PTC-100™ programmable thermal controller, MJ. research Inc) under the following condition: initial denaturation(5 min at 94°C) followed by 40 cycles of denaturation (30 sec at 94°C), annealing(30 sec 62°C) and extension(30 sec at 72°C). A final extension step was carried out for 5 min at 72°C, and 4°C forever. Through many experiments the optimal final concentration of primers in triplex PCR reaction was determined. The PCR condition and concentration of each component for 16S rDNA gene referred to the previous study [24].

### Construction of recombinant plasmid as standard template

DNA templates were extracted from standard strains of *E. coli *O157:H7 W933 and *Vibrio cholerae *O139 M045 respectively, which were amplified using conventional PCR with unlabelled primers. The PCR products were purified, and then inserted into pGEM-T vectors (Promega). Consecutively, the recombinant products were transformed into *Escherichia coli *DH5α (Promega), and incubated overnight at 37°C with shaking. Recombinant plasmids were screened using the T7 and SP6 primers and extracted using Promega Plasmid Extracting Kit, which procedure was according to the manual. After purification, the insert DNA fragments were identified through sequencing using automatic DNA sequencer (CEQ-2000). The number of plasmid copies was counted as previously described [25]. The plasmid was 10-fold serial diluted and stored at -20°C for use as PCR template.

### Preparation of the oligonucleotide microarray

The 3' end amino-modified probes were diluted to a final concentration of 50 μmol/L in spotting solutions (3 × SSC and 0.01% SDS) and were transferred into 384-well micro-titer plates in volumes of 10 μL. The probe solutions were spotted to aldehyde-coated glass slides (CEL Associates) with a microarray printer (Cartisan), which deposits 0.5 nL at each spotting site, resulting in spots of 200 μM in diameter. The humidity during spotting was 90% and the temperature kept at 23°C. After spotting, slides were incubated for another 2 h under the same conditions and stored at room temperature for at least 24 h before use.

### Hybridization and signal detection

The Cy3-labeled PCR products were mixed with the hybridization solution (6 × SSC, 1 × Denhard, 0.2% SDS), and 10 μL of the hybridization solution was transferred to the hybridization area on the glass slide. The slide was incubated in 42°C water bath for 1 h in a tightly-sealed hybridization chamber. After incubation, the slide was washed sequentially in washing solution A (1 × SSC, 0.2% SDS), washing solution B (0.2 × SSC) and washing solution C (0.1 × SSC) for 1 min each.

The glass slides were scanned using the GenePix 4000B (Axon), with excitation at 540 nm and emission at 570 nm (Cy3). Sixteen-bit TIFF images of 10 micrometers resolution were analyzed with software GenePix Pro 4.0. The signal intensity is displayed according to spot color, whose order from high to low is white, red, yellow, green, and blue. After the removal of local background, the average signal intensity of each probe was calculated. The cutoff value was evaluated in order to confirm whether the target was considered to be positive or not.

### Assay sensitivity

The sensitivity was evaluated according to serial dilution (10-fold) from 10^4^~10^1 ^copies/μL. Meanwhile, according to Jin et al[24], the sensitivity about the amount of bacteria in clinical specimen was determined. Each dilution series of genomic DNA were mixed with a natural background of genomic DNA from stool samples of healthy volunteers. The genomic DNA of stool samples was extracted as shown above. After the whole operation, two kinds of sensitivity were determined through signal intensity according to cutoff value. Additionally, the sensitivity of real time PCR (In real time PCR, the fluorescent probe detected the same unique region in *LPS*gt gene as the detecting probe of the oligonucleotide microarray) (DaAn Gene Co., Ltd, Guangzhou, China) and conventional microbiological methods was determined in order to compare the sensitivity of this assay with other methods.

### Statistical analysis

Statistical analysis in this paper was made with SPSS for Windows software (version 11.5, Chicago, Il, USA).

## Authors' contributions

The author's current institute is Zhejiang Provincial Center for Disease Control and Prevention, Hangzhou, China. Tel/Fax: 86-571-87235092; E-mail: dazhijin@163.com.

The authors wish it to be known that, in their opinion, the first two authors should be regarded as joint First Authors.

D-ZJ and X-JX carried out the all the experiments; S-HC participated in the design of oligonucleotide mocriarray; S-YW drafted the manuscript; ZZ and FL collected the clinical specimens; X-EM performed the statistical analysis; S-QW designed and conceived of all the study and participated in its coordination. All authors read and approved the final manuscript.
